# Job Preferences of Nurses and Midwives for Taking Up a Rural Job in Peru: A Discrete Choice Experiment

**DOI:** 10.1371/journal.pone.0050315

**Published:** 2012-12-20

**Authors:** Luis Huicho, J. Jaime Miranda, Francisco Diez-Canseco, Claudia Lema, Andrés G. Lescano, Mylene Lagarde, Duane Blaauw

**Affiliations:** 1 School of Medicine, Universidad Peruana Cayetano Heredia, Lima, Peru; 2 School of Medicine, Universidad Nacional Mayor de San Marcos, Lima, Peru; 3 Instituto Nacional de Salud del Niño, Lima, Peru; 4 CRONICAS Centre of Excellence in Chronic Diseases, Universidad Peruana Cayetano Heredia, Lima, Peru; 5 Salud Sin Límites Perú, Lima, Peru; 6 Department of Parasitology, and Public Health Training Program, US Naval Medical Research Unit 6 (NAMRU-6), Lima, Peru; 7 School of Public Health and Administration, Universidad Peruana Cayetano Heredia, Lima, Peru; 8 Department of Global Health and Development, Faculty of Public Health and Policy, London School of Hygiene and Tropical Medicine, London, United Kingdom; 9 Centre for Health Policy, School of Public Health, Faculty of Health Sciences, University of Witwatersrand, Johannesburg, South Africa; World Health Organization, Switzerland

## Abstract

**Background:**

Robust evidence on interventions to improve the shortage of health workers in rural areas is needed. We assessed stated factors that would attract short-term contract nurses and midwives to work in a rural area of Peru.

**Methods and Findings:**

A discrete choice experiment (DCE) was conducted to evaluate the job preferences of nurses and midwives currently working on a short-term contract in the public sector in Ayacucho, Peru. Job attributes, and their levels, were based on literature review, qualitative interviews and focus groups of local health personnel and policy makers. A labelled design with two choices, rural community or Ayacucho city, was used. Job attributes were tailored to these settings. Multiple conditional logistic regressions were used to assess the determinants of job preferences. Then we used the best-fitting estimated model to predict the impact of potential policy incentives on the probability of choosing a rural job or a job in Ayacucho city. We studied 205 nurses and midwives. The odds of choosing an urban post was 14.74 times than that of choosing a rural one. Salary increase, health center-type of facility and scholarship for specialization were preferred attributes for choosing a rural job. Increased number of years before securing a permanent contract acted as a disincentive for both rural and urban jobs. Policy simulations showed that the most effective attraction package to uptake a rural job included a 75% increase in salary plus scholarship for a specialization, which would increase the proportion of health workers taking a rural job from 36.4% up to 60%.

**Conclusions:**

Urban jobs were more strongly preferred than rural ones. However, combined financial and non-financial incentives could almost double rural job uptake by nurses and midwifes. These packages may provide meaningful attraction strategies to rural areas and should be considered by policy makers for implementation.

## Introduction

There is wide agreement on the need of evidence-based interventions to adequately face the health workforce crisis that affects health systems, particularly in developing countries [1]–[3]. Such interventions should be designed, implemented and evaluated with the support of a sound body of knowledge. Yet, what is good evidence is harder to agree on [4]–[6]. A critical aspect to answer is how we can reliably identify those incentives that would actually persuade health workers to work in remote and rural underserved areas.

Discrete choice experiments (DCE) have recently been applied to the field of human resources of health (HRH), particularly to identify attraction and/or retention incentives for health care workers. DCE is a well-suited method to stated job preferences of health workers, given specified attributes and levels [7], [8]. DCE studies can provide information on which specific job attributes are stronger and which are weaker. The policy relevance of the resulting preferences may depend not only on how strong the particular choices are, but also on how realistic they are from policymakers' and health workers' perspectives, and on the context-specific characteristics of the labour market.

Recently, the Peruvian Ministry of Health has led the planning of Prosalud [9], a strategy aimed at increasing the presence of basic health teams – doctors, nurses, midwives and nurse technicians – at primary care and secondary level across the country, with priority given to rural and poorest areas. Prosalud has been conceived to complement scaling-up efforts of a wider health system reform (Universal Health Insurance, Aseguramiento Universal en Salud) aimed at providing universal health services. Success of this health reform depends heavily on an effective attraction and retention HRH strategy. Whilst Prosalud program considers a series of incentives, their relative strength has not been systematically explored.

A DCE study was conducted to identify the relative strength of various incentives, including those currently being promoted by Prosalud, which may stimulate nurses and midwives currently on a short-term contract basis to work in rural areas. Lessons learned from such an exercise would be useful for better-informed policy planning and implementation of HRH interventions at the national and local level in Peru, and would be of interest to other similar settings in the international context.

## Methods

### Ethics Statement

The study protocol and the informed consent forms were approved by the Ethics Committee of Universidad Peruana Cayetano Heredia, Lima, Peru, by the Ethics Review Committee of the World Health Organization, Geneva, and by the Institutional Review Board of the US Naval Medical Research Unit No. 6 (NAMRU-6), Lima, Peru. Participation was voluntary and all participants signed the informed consent form before any study procedure.

### Peruvian context: the health workforce

Peru, recently ranked as an upper-middle-income economy [10], hosts an inequitable health workforce distribution and is one of the few countries in Latin America considered to have a HRH crisis [11]. The maldistribution not only affects doctors but also other health cadres such as nurses and midwives [12], [13]. Recent studies on HRH in Peru have shown a largely heterogeneous labour market, with different health worker cadres and diverse job regimes [12], [13], in particular in the public sector. Lack of a clear career pathway based on merit, low salaries, lack of motivation, as well as dual practice in both public and private sectors, complete the health labour landscape in the country [12]–[14].

Within the public sector's health workforce, two clearly contrasting job regimes dominate the Peruvian labour market. Firstly, a stable job (*nombrados*) that includes a permanent post with various labour benefits including paid holidays, social security covering health care, as well as a retirement fund, among the main ones. Secondly, a temporary job under a contract (*contratados*) of variable duration, usually of one year but may be less (three-month contracts are not uncommon) which can be withdrawn at any time by the employer, and the absence of the benefits described for stable positions [15].

The most frequent short-term contract schemes in place at the time of this study included SERUMS – a graduate public health rural service with one year duration, RECAS – a contract of 3–6 months duration that can be renewed at the employers' discretion, and CLAS – a contract with Ministry of Health facilities -managed by communities. Under these different contract schemes, nurses and midwives can work directly for the Ministry of Health or indirectly through social or development strategies, but all employed ultimately by the public sector.

By 2007, 13,275 doctors, 13,228 nurses and 6,531 midwives worked at the Peruvian Ministry of Health. Of this total, 60.5% were *nombrado*s, while the remaining proportion were *contratados* [16]. The proportion of *contratados* was 17% for doctors, 45% for nurses and 67% for midwives.

### Study setting and study population

Ayacucho department is located in the south Andean region of Peru, and it is one of the poorest departments of the country. It is politically divided into provinces, and each province into districts. Ayacucho city, the department's capital, and the capitals of provinces constitute the urban areas, while most inner and remote districts are rural areas.

Ayacucho was the core area of social unrest and political violence that affected Peru during the 1980s and 1990s, and it is still struggling with the subsequent recovery process [17], [18]. Although the proportion of people living in rural areas have been declining progressively over time (as shown in the related paper on doctors), recent figures indicate that 58.9% of its population live in rural areas [19]. A substantial proportion of high maternal and child mortality is related to scarcity of capable and motivated health workers in Ayacucho [20], [21].

The density of nurses in Lima – the capital city of Peru - is 3.59 per 10,000 population, whereas in Ayacucho is 3.33 [16]. Density figures for midwives are 0.38 and 0.68 per 1,000 women of reproductive age in Lima and Ayacucho, respectively. However, most nurses and midwives within Ayacucho are still employed in the capital city (Ayacucho), with a substantial proportion of primary level facilities in rural and remote areas lacking their presence [16]. Additionally, nurses and midwives in urban Ayacucho can also work for the private sector, while this possibility in rural areas is largely unfeasible.

As in other departments of the country, the Ministry of Health is largely responsible for providing health care in Ayacucho. The local health system specifically comprises a regional hospital located in the capital city; health centers, most of them located in the periphery of Ayacucho city and in other capital departments; and health posts, mainly located in rural and remote areas of the department.

The study was conducted in the poorest districts of Ayacucho, with a human development index (HDI) equal to or lower than 0.5074, and located in seven provinces located in the northern part of the department. Details on sociodemographic information of the study provinces are shown in Table 1.

**Table 1:** Sociodemographic and health access characteristics of the seven Ayacucho provinces selected for the study.

	Rural/urban proportion	Annual population growth rate, 1993–2007 (%)	Illiteracy rate (%)	Illiteracy rate, rural areas (%)	Proportion of adolescent mothers (%)	Population with Quechua as native language (%)*	Households with safe drinking water (%)	Households with electricity (%)	Population without any health insurance system (%)
**Huamanga**	0.37	2.7	12.7	28.3	11.9	51.3	90.8	70.8	45.5
**Huanta**	1.18	2.8	21	28.6	19.8	68.2	91	43.5	50.2
**La Mar**	1.45	2	24.1	27.8	25.6	83.6	87.3	25.4	49.3
**Vilcashuaman**	2.15	1.5	26.2	30.4	19.5	90.3	83.7	18.7	46.7
**Huancasancos**	0.48	1.8	18.3	25.6	12.4	81.8	66.1	41.7	30.7
**Cangallo**	1.87	1.5	26.7	29.9	15.1	90.6	92	33.9	30.7
**Victor Fajardo**	0.34	1.3	22.5	29.7	13.5	87	91	55.4	34.8

* Learned during infancy/childhood.

Source: National Institute of Statistics and Computing (INEI) - Population and Household National Censuses, 1993 and 2007.

Nurses and midwives working on a short-term contract basis for the Ministry of Health in Ayacucho were the target group of this study. From a programmatic perspective, any attraction or retention intervention targeting nurses and midwives needs to include both those working in an urban area and those already working in a rural setting. Of course, attraction would be the policy relevant issue for the group located in the urban area, while retention would be the goal for those already working in rural health services. We therefore included in our study both urban and rural nurses and midwives.

### Sampling

A cluster sampling method was used with sampling of facilities in proportion to the distribution of health facilities, and targeted personnel in the study area. Prior to the initiation of field activities, each health micro-network in the selected districts was visited and a preliminary list of all eligible health personnel was compiled to serve as the sampling frame. This activity was performed to overcome the lack of updated and reliable information on the actual number and location of health workforce at both central and local levels, so as to assemble an adequate sampling frame.

Based on the experience of previous studies [7], [22], we aimed for a sample size of 80 nurses and midwives working on a short-term contract basis in urban areas and another 80 working in rural areas. An extra 25% was added to account for potential rejections to participate in the study or to consider that a fraction of the personnel would not be in their work sites at the times of the fieldwork. Reaching this sample size required the random selection of 82 health facilities. All nurses and midwives in the selected facilities were invited to participate in the study.

### Discrete choice experimental design

The identification of the most relevant attributes and their possible levels relied on several methods: in-depth interviews and focus groups with nurses and midwives, review of the international literature on attraction and retention strategies in low- and middle-income countries, and interviews with policy makers. The final DCE attributes and their levels were defined through iterative group discussions among the research team members (interviewers, analysts, researchers and DCE technical advisors).

We opted for a labelled discrete choice design. The two labels of interest corresponded to the two main geographic areas where nurses in our study could be posted: ‘Rural community’ *and* ‘Ayacucho city’. The labelled design was chosen because it allows researchers to define different attributes and levels for the different labels, thus increasing the realism of the task and making it possible to define specific incentives for a particular geographic area [7], [23], [24].

A set of 8 attributes were identified as potential determinant factors for nurses when choosing a job in a rural or urban setting (Table 2): type of facility in which they could be posted, monthly net salary, number of years they would have to work in the post before getting a permanent contract (‘nombramiento’), bonus points when applying for specialist training, a scholarship for specialist training, provision of free housing, expected work schedule (excluding holidays), and certificate of recognition of rural service.

**Table 2:** Final DCE design.

	RURAL COMMUNITY	AYACUCHO CITY
**1. Health facility**	• Health post• Health center	• Health center• Regional hospital
**2. Monthly take home (after tax) salary**	• S/. 1,000• S/. 1,250• S/. 1,500• S/. 1,750	• S/. 1,000
**3. Time in post before getting permanent job**	• 3 years• 6 years	• 6 years• 10 years
**4. Points when applying for training in Family and Community Health Specialization, after 3 years in post**	• 10 points bonus when applying for training in Family and Community Health Specialization• 20 points bonus when applying for training in Family and Community Health Specialization	• None• 10 points bonus when applying for training in Family and Community Health Specialization
**5. Scholarship for training in Family and Community Health Specialization, after 3 years in post**	• No• Yes	• No
**6. Free housing provided**	• A shared room in a residence with shared facilities• A 2-bedroomed independent house	• None
**7. Work Schedule (excluding holidays)**	• You work 22 days and then have 8 days off• You work 18 days and then have 12 days off	• You work everyday except Sundays
**8. Recognition of rural service**	• No• You get an official certificate of recognition	• No

The salary attribute had four levels to allow for evaluation of nonlinear effects. All other attributes had two levels, which resulted in a full factorial design with 4,096 combinations (i.e.2^10^×4^1^). We used the macros developed by Kuhfeld [25] for SAS (SAS, Cary, NC, United States of America) to select combinations for an orthogonal main effects design, and to organize the selected profiles into the most D-efficient choice design.

Once the DCE design was defined, the resulting tools were piloted twice prior to field application, first in Lima with health professionals, and then in Huancavelica, an area similar and close to Ayacucho. Following these pilots, changes were made to the wording of the levels and attributes. The resulting final design is shown in Table 1.

The DCE questionnaire was in Spanish and had 16 choice tasks. Respondents were asked to choose one of the two alternatives offered or could decide to stay in their current job (‘opt out’). If they chose to opt out, they were then presented with a forced choice where they had to make a choice between the two proposed jobs. This was done to limit the potential loss of information if a high proportion of respondents chose to opt out.

An additional questionnaire was developed to collect information on the socio-demographic characteristics of respondents that were thought to be influential of job choices and the characteristics of their current job. The field team explained and administered the DCE and the socio-demographic questionnaires to the participants.

### Statistical analysis

To provide a brief description of the population study, simple statistical tests were used to compare the midwives and nurses.

Considering the small proportion of respondents who chose the third (opt-out) option, we only analysed the responses of the forced choice questionnaire. We used multiple conditional logistic regressions to evaluate the importance of job attributes and of individual socio-demographic characteristics on job preferences. We compared the relative importance of attributes through calculation of odds ratios and their confidence intervals, while the preferences of different subgroups were evaluated by including interaction terms in the regression models. Following this analysis, we used the best-fitting estimated model to predict the impact of potential policy incentives on the probability of choosing a rural job or a job in Ayacucho city.

We conducted all analyses with Stata 11.0 for Windows (Stata Corp., College Station, TX, 2010).

## Results

Overall 205 contract nurses and midwives participated in the study. Basic socio-demographic characteristics of participants are shown in Table 3. In brief, the study sample of nursing and midwifery's health workers were mostly in their early thirties, predominantly female, almost two thirds were native to Ayacucho, and a similar proportion working in urban and rural areas. On average, they had already worked for 2.4 years in a rural area.

**Table 3:** Basic sociodemographic characteristics of participants.

	Number	Percentage
**Age in years:** mean (sd)	32.5 (6.4)	
**Birth place**		
Ayacucho	129	62.9
Ica	12	5.9
Lima	26	12.7
Other	38	18.5
**Gender**		
Male	18	13.7
Female	177	86.3
**Current job area**		
Urban	98	47.8
Rural	107	52.2
**Children**		
Yes	100	48.8
No	105	51.2
**Years of professional experience:** mean (sd)	4.5 (3.5)	
**Years of experience in rural area:** mean (sd)	2.4 (2.7)	

Table 4 summarizes the results of the final conditional logit model comparing the impact of different attributes on the odds of choosing a rural job against the odds of choosing an urban post. Considering the baseline levels of all attributes, there seemed to be a strong preference for jobs in urban areas. For example, the label Ayacucho city was 14.74 times more likely to be chosen compared to the odds of choosing a rural setting.

**Table 4:** Determinants of job preferences for nurses and midwives on a short-term contract.

	Odds Ratios	95% CI	p-value
**Alternative-specific constant**			
Ayacucho city	**14.74**	**4.97; 43.73**	**<0.001**
**Rural job characteristics**			
Health center vs. health post	**1.18**	**1.02; 1.38**	**0.03**
Salary increase - per each S/. 1,000 nuevos soles	**2.95**	**2.25; 3.88**	**<0.001**
Years before getting permanent job - per each year	**0.95**	**0.90; 1.00**	**0.05**
Specialization - per each 10 points	1.02	0.87; 1.18	0.824
Scholarship vs. no scholarship	**1.16**	**1.00; 1.35**	**0.05**
Independent house vs. shared room	1.01	0.87; 1.17	0.912
Days of work per month – per extra working day	1.01	0.98; 1.05	0.512
Rural recognition certificate vs. no certificate	1.03	0.89; 1.20	0.69
**Urban job characteristics**			
Regional hospital vs. health center	1.07	0.92; 1.24	0.413
Years before getting permanent job - per each year	**0.92**	**0.89; 0.96**	**<0.001**
Specialization - per each 10 points	1.12	0.96; 1.30	0.148
**Socio-demographic characteristics**			
Male	**1.74**	**1.37; 2.20**	**<0.001**
Birthplace (Urban Ayacucho vs. outside Ayacucho)	0.97	0.81; 1.16	0.713
Birthplace (Rural Ayacucho vs. outside Ayacucho)	**3.27**	**2.25; 4.74**	**<0.001**
Does not live with partner vs. does not have a partner	**0.71**	**0.58; 0.86**	**0.001**
Lives with partner vs. does not have a partner	0.98	0.79; 1.22	0.883
Years of experience, 2–4 vs. <2 yrs (2nd. vs. 1st quartile)	0.85	0.65; 1.12	0.247
Years of experience, 5–7 vs. <2 yrs (third. vs. 1st quartile)	**0.48**	**0.35; 0.66**	**<0.001**
Years of experience, 8–14 vs. <2 yrs (fourth vs. 1st quartile)	**0.60**	**0.43; 0.82**	**0.002**
Midwife vs. nurse	**0.77**	**0.66; 0.90**	**0.001**
Paid SERUMS vs. other (temporary or permanent)	0.94	0.70; 1.24	0.647
Salary within or above the offered range	**2.15**	**1.79; 2.58**	**<0.001**
Hospital vs. health post/center	**0.22**	**0.18; 0.27**	**<0.001**
Has children	1.13	0.92; 1.38	0.235
Currently studying diploma/MSc/PhD/Specialization	1.06	0.90; 1.26	0.47
Workload scale (1–10)	1.02	0.97; 1.06	0.446
Likely to remain in the current post for another year	**1.83**	**1.55; 2.16**	**<0.001**
**N**	**205**		

Pseudo R^2^: 0.1442 Log-likelihood: −1945 Chi^2^ (28) = 655 p<0.001.

For rural job attributes, those that influenced significantly the odds of choosing a rural post included a salary increase of PEN 1,000 soles (OR 2.95, p<0.0001), health center *versus* health post (OR 1.18, p = 0.03), scholarship *versus* no scholarship (OR 1.16, p = 0.05), and years before getting a permanent post (OR 0.95, p = 0.05) that acted as a disincentive. All the remaining attributes were not significant, including bonus points when applying for specialist training, scholarship for specialist training, provision of free housing, monthly workload, and certificate of recognition of rural service. For urban job attributes, only time before getting a permanent job was significant, acting also as a disincentive (OR 0.92, p<0.0001).

The influence of socio-demographic characteristics was explored through interaction with rural label. Male gender, rural place of birth, having a salary within or above the offered range, and the likelihood of remaining for another year in current post increased significantly the chances of choosing a rural job. Conversely, not living with a partner, having accumulated 5–7 years or 8–14 years of work experience, being a midwife rather than a nurse, and working at a hospital rather than at a health post or health center decreased significantly the likelihood of choosing a rural job (Table 4). All the remaining socio-demographic factors were not significant.

**Figure 1:**
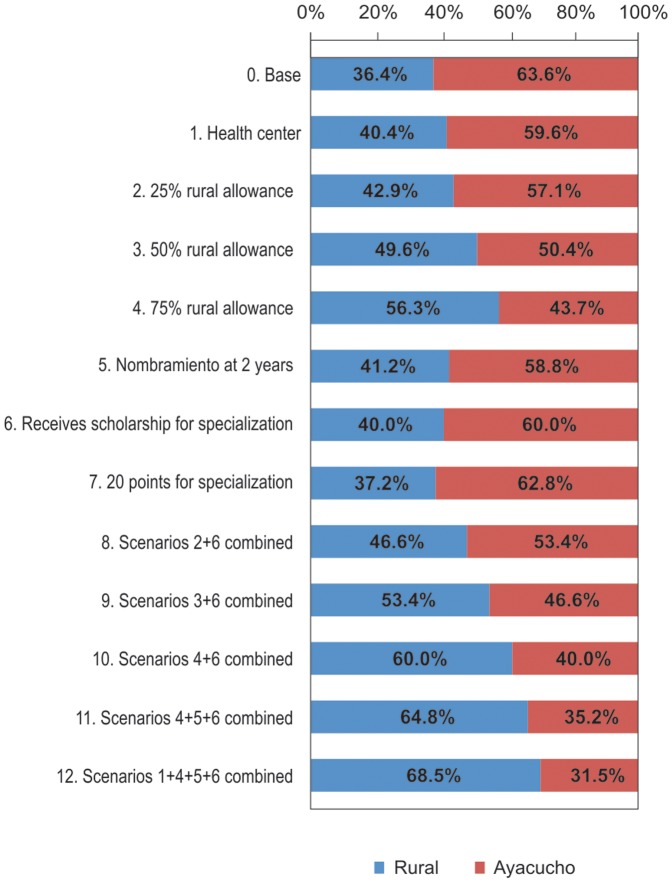
Policy simulations showing changes in proportion of health workers opting for a rural job when individual or combined incentives are offered, relative to base scenario*. *The scenarios correspond to simulations, when individual or combined incentives could be offered, relative to baseline scenario and using the coefficients of each specific attribute studied.

The results of the conditional logistic model were used to simulate the effect of different policy incentives, alone or in combination, on the proportion of nurses and midwives who would choose a rural job. These simulations were conducted under realistic base scenarios, one for rural and one for urban setting, prevailing at the time of the study (Figure 1). These scenarios were as follow: A) Rural community: health post, salary S/. PEN 1,000 nuevos soles, permanent job granted after 6 years, no points for specialization, no scholarship for specialization, free housing provided (as specified in Table 2); and, B) Ayacucho city: regional hospital, salary S/. PEN 1,000 nuevos soles, permanent post granted after 6 years, no points for specialization, no free housing.

Figure 1 displays the results of the simulations. Under the base scenario, it was estimated that only 37% of nurses and midwives would choose a rural job. This percentage increased to 40.4% when health center was added as an attribute, to 42.9%, 49.6% and 56.3% when rural allowance was increased by 25%, 50% and 75%, respectively. Inclusion of getting a permanent position as an isolated attribute would persuade a total of 41.2% of nurses to take a rural job, while 40% and 37.2% would be persuaded if they were offered a scholarship for specialization, or 20 points as a bonus to apply for a specialization, respectively. The percentage of nurses who would choose a rural job reached 60% when a 75% salary increase and provision of scholarship for specialization were considered together. Adding the attribute of getting a permanent position after two years to the previous combination would persuade 64.8% of nurses to choose a rural job. Finally, 68.5% of nurses would likely choose a rural job if they were offered a combination of health center, 75% rural allowance, getting a permanent job after two years, and a scholarship for specialization.

## Discussion

In our study population, strong preferences of nurses and midwives for taking an urban job in contrast to a rural one were observed. In general, the different individual incentives included in the study were not powerful enough to persuade a majority of nurses and midwives to opt for a rural job, except for a substantial salary increase. These findings pose major challenges for planning human resources policies aimed at prioritizing underserved areas. Any policy implemented in this or similar settings will have to compete with a strong urban preference of 14.7 times over a rural job.

As we showed in a related qualitative study, doctors, nurses, midwives and nurse technicians incentives all feel that the rural setting is clearly a disadvantageous place to remain for themselves and their families, and they therefore considered it as a place to stay only transiently, while waiting for better job opportunities [26].

The lack of a significant effect of the assessed job attributes as potential incentives alone suggest that they are too weak for the expectations of nurses and midwives working on a temporary basis for the public sector in Peru.

However, we also found that certain socio-demographic characteristics of the studied population might modify significantly the odds of choosing a rural post against the odds of choosing an urban one. Specifically, being a male health worker born in a rural area, with a salary within or above the offered range and the expressed likelihood of remaining in current post increased significantly the chances of choosing a rural position. Conversely, being a midwife, living with a partner, having accumulated several years of job experience, and holding a post at a hospital decreased significantly the likelihood of choosing a rural job. These are aspects that need to be taken into account when planning an attraction or retention strategy.

Also, different combinations of incentives explored through simulations revealed to have potential for influencing the chances of choosing a rural post.

Our findings are in agreement with results of other studies performed in other developing countries, highlighting the need to combine financial and non-financial incentives [7], [8], [27]. They also support the WHO recommendations that emphasize the implementation of bundle interventions rather than individual incentives [6]. However, they also show the limitations of currently proposed policy incentives in terms of their relative importance within the context of a rural area strongly perceived as an unfavourable setting to live and work in, where there are scarce opportunities for personal, family and professional development.

Our results need to be interpreted within the framework of training characteristics of nurses and midwives, of the health labour market prevailing at the time of the study [28], [29], and within the context of wider health reforms related to access to health services and to the health workforce distribution.

In contrast to training of doctors which is dominated by clinical content, the training of nurses and midwives, although also clinically-oriented, includes a significant community-oriented component that offers them promotional and preventive approaches to public health problems [30], [31]. On the other hand, the scope of practice for nurses and midwives is quite different than that for doctors. Although specialization courses may offer them the opportunities for working at referral facilities, the number of the available clinical posts is limited [30], [31]. Their inclusion in interventions focused on primary health care such as maternal and child health and communicable diseases is an alternative scope of practice. Within this scenario, although the majority of nurses and midwives may still prefer a clinical urban post, they would be persuaded to take a rural job if this offers incentives strong enough to counter the perceived flaws of the rural setting. Actually, many primary level managerial and clinical posts are filled in by nurses and midwives rather than by doctors [30], [31].

One additional labour market factor that may persuade short-contract nurses and midwives to work in a rural position if sufficiently attractive incentives are offered, may be related to the fact that *contratados* are most commonly younger health workers recently incorporated to the labour market, while *nombrados* are older, and have generally been working for longer periods before they were granted their current stable job condition [12], [16]. Also, *contratados* are more likely to be single and with lesser family commitments than *nombrados* [12], [16]. Moreover, the common aspect to the various types of short-term contract is that health workers could work without a formal and permanent link to the health system [12], [16]. Unfortunately, this flexibility for hiring professionals for short-term periods is also related to job instability expressed by the fact that employees can be fired at any moment or may lose their job when their contract period ends [12], [16]. Therefore *contratados* may be willing to accept rural posts if they feel that in this way they will assure a position for a few years, as revealed by our related qualitative study [26]. Our current DCE also showed that lengthening the waiting time before getting a permanent post acted as a significant disincentive factor, although it was not particularly powerful, and therefore that decreasing this waiting time would act as a an incentive, a point we also discuss in the section on policy simulations.

On the other hand, Prosalud, although not yet implemented, includes a variety of incentives for members of basic health teams – doctors, nurses, midwives and nurse technicians – aimed at improving their deployment in remote rural areas, mainly at primary and secondary care level [9]. The main incentives considered by Prosalud are the provision of bonus points when applying to a public scholarship for training in family and community health specialization courses, after completion of three years in a rural post [9].

Prosalud builds upon SERUMS, which has been in place in Peru for several decades [9]. Based on this established strategy for deployment of rural health workers, Prosalud aims at extending the current one-year rural SERUMS placement to up to three years. In addition to the above-described non-financial incentives, Prosalud also considers incorporating other incentives including differential salary and payment scales, improved housing facilities, and continuous professional development programs, among others [9].

The policy simulations we performed started with urban and rural baseline scenarios trying to reproduce prevailing conditions at the time of the study and then included various individual and combined incentives. Progressive changes of attributes were made to assess the extent of change in the proportion of nurses and midwives choosing a rural job. These changes included the incentives planned for Prosalud. This exercise therefore provided useful information for refining the incentives of this particular strategy and further for planning other attraction and retention strategies.

Firstly, barely a third of nurses and midwives would choose a rural post under the base scenario for a rural community. This proportion would increase to about half if a 50% of salary increase was offered, and up to 56% if the rural allowance offered would increase by 75%. Although currently they represent significant improvements in health workforce deployment, such isolated salary incentives may not have the same magnitude of effect on attraction and retention in the future. Actually, health workers' salaries have been progressing over the years, even if they have not reached yet competitive amounts [12], and they will very likely increase in greater proportion in a near future, as universal health insurance is scaled-up and deployment of health workforce as a crucial bottleneck becomes more pressing. Moreover, salaries may also increase as a consequence of collective negotiations with health professionals' trade unions [16], [28].

Secondly, shortening by two years the waiting time before getting a permanent post would increase the proportion of nurses and midwives choosing a rural post to 41%. This isolated incentive does not seem comparatively very attractive, although it is a claim consistently raised by trade unions whenever they ask for improvement of labour conditions. It may seem fiscally feasible under the current Peruvian conditions of economic growth, but it may prove to be hard to sustain in the long-term, particularly if the production of nurses and midwives by academic institutions is substantially increased.

Thirdly, the provision of a scholarship for following a specialization or granting points when applying for specialization training courses were not particularly strong incentives either, and thus they should be considered as areas needing reconsideration and strengthening when designing attraction and retention strategies like Prosalud.

Finally, combining attributes was more effective than individual interventions. The most effective package included a 75% of salary increase plus provision of a scholarship for specialization, which would increase the proportion of nurses and midwives choosing a rural post from about a third to 60%. The combination of a substantial salary increase, access to a permanent position after a reduced waiting time (two years) and provision of a scholarship would increase the proportion choosing a rural job to 65%, while adding the offer of a job in a rural health center to the former combination would increase the proportion to almost 69%. However, possible drawbacks of these last two combinations should be carefully considered. They would risk posing a substantial burden on the central and local government fiscal balance and on the human resources management system, and therefore it should be considered whether they would be acceptable by the ministry of finance and by the health sector policymakers. It should also be considered whether they are perceived as unrealistic for nurses and midwives themselves. An ideal combination of incentives including all possible factors would require a huge commitment from the government, and thus appears to be challenging in the current circumstances, due to the burden that would pose on human and fiscal resources.

Thus according to the characteristics of the health labour market in Peru [28], [29], nurses and midwives working on a short-term contract basis would be more likely to be persuaded to take a rural post by combinations of incentives that include substantial salary increases along with clear and realistic opportunities for postgraduate training.

We need to acknowledge some limitations of our study. First, we cannot anticipate with certainty whether participants will actually make the decisions they stated in the study. Several intrinsic factors and external interacting factors present in real life can affect the actual job choice decisions. Therefore, the actual impact of the attraction and retention strategies can only be captured fully through longitudinal studies that are able to show whether the different health cadres actually make the decision predicted by the policy simulations. Second, the results of our study can be applied to the labour market conditions prevailing in Peru at the time of the study, which can evolve over time, and therefore updated analyses may be needed to avoid under-emphasis or over-emphasis of any given attribute, or to introduce new ones that may seem warranted. Third, our results represent the stated preferences of nurses and midwives working on a short-term contract, and they could not have captured incentives particularly relevant to those with a permanent position.

The World Health Organization (WHO) has recently developed health worker retention recommendations, with particular emphasis on developing countries, which are those with the highest need of capable and motivated health workers [6]. Specific categories of recommendations include: a) education, b) regulatory, c) financial incentives, and d) personal and professional support. Although they have been developed through an extensive literature review and a wide consultation and debate process with experts from all regions of the world, the recommendations are in general based on weak evidence, and furthermore, they do not provide information on the relative strength of each individual intervention, or of components of combined interventions. Our study contributed to filling this gap, although it must be emphasized that the relative strength of incentives might vary from one setting to another.

In conclusion, urban jobs were more strongly preferred than rural ones. Combined financial and non-financial incentives could almost double rural job uptake by nurses and midwifes. These packages may provide meaningful attraction strategies to rural areas and should be considered by policy makers for its implementation, while weighing carefully their feasibility and sustainability.
